# Intraspecific variability in thermal tolerance: a case study with coastal cutthroat trout

**DOI:** 10.1093/conphys/coac029

**Published:** 2022-05-12

**Authors:** Kara Anlauf-Dunn, Krista Kraskura, Erika J Eliason

**Affiliations:** 1 Oregon Department of Fish and Wildlife, 28655 Highway 34, Corvallis, OR 97333, USA; 2 Department of Ecology, Evolution, and Marine Biology, University of California Santa Barbara, Santa Barbara, CA 93106, USA

**Keywords:** thermal tolerance, physiology, metabolism, intraspecific, cutthroat trout, Climate change

## Abstract

Fish physiological performance is directly regulated by their thermal environment. Intraspecific comparisons are essential to ascertain the vulnerability of fish populations to climate change and to identify which populations may be more susceptible to extirpation and which may be more resilient to continued warming. In this study, we sought to evaluate how thermal performance varies in coastal cutthroat trout (*Oncorhynchus clarki clarki*) across four distinct watersheds in OR, USA. Specifically, we measured oxygen consumption rates in trout from the four watersheds with variable hydrologic and thermal regimes, comparing three ecologically relevant temperature treatments (ambient, annual maximum and novel warm). Coastal cutthroat trout displayed considerable intraspecific variability in physiological performance and thermal tolerance across the four watersheds. Thermal tolerance matched the historical experience: the coastal watersheds experiencing warmer ambient temperatures had higher critical thermal tolerance compared with the interior, cooler Willamette watersheds. Physiological performance varied across all four watersheds and there was evidence of a trade-off between high aerobic performance and broad thermal tolerance. Given the evidence of climate regime shifts across the globe, the uncertainty in both the rate and extent of warming and species responses in the near and long term, a more nuanced approach to the management and conservation of native fish species must be considered.

## Teaser

Coastal cutthroat trout (*Oncorhynchus clarki clarki*) are wide-ranging species, distributed throughout the river network. In this study, we evaluated how thermal performance varied in coastal cutthroat trout across four hydrologically distinct watersheds. Coastal cutthroat trout displayed considerable variability in physiological performance to varying temperature treatments. Understanding species physiological responses to current thermal regimes allows for a more nuanced approach to the management and conservation of native fish species into the future.

## Introduction

Shifts in climate regimes are projected to be associated with both changes in mean conditions as well an increase in the variability and frequency of extreme events (Bernhardt *et al.*, 2020). The capacity for fish to cope with temperature change will be primarily mediated by their adaptive capacity and phenotypic plasticity (Seebacher *et al.*, 2015b; Bernhardt *et al.*, 2020). An understanding of the physiological tolerance of fish to changes in environmental temperatures is essential when trying to evaluate how climate change will influence biogeographic distributions and survival (Harada *et al.*, 2019; Sunday *et al.*, 2012; Walters *et al.*, 2018). Monitoring stream temperature and evaluating components of the thermal regime (e.g. daily variability and range, duration of warm events, etc.) are valuable to understanding current and future shifts in stream temperatures (Arismendi *et al.*, 2013). However, without complementary information relating thermal data to species thermal habitat requirements, we will be limited in our ability to make decisions about how best to manage and protect species in general, and more specifically those species most at risk (Marras *et al.*, 2015; Zillig *et al.*, 2021). Conservation and management of fish in a changing climate will depend on knowledge about both the rate and magnitude of change in the aquatic environment and the effects on fish physiology (Marras *et al.*, 2015). The physiological response of fish to a changing climate provides a mechanistic explanation for population responses such as altered phenology, range shifts and biotic interactions.

Physiological performance traits (e.g. metabolic rates, locomotion, digestion, growth) of fish and other ectotherms are temperature-dependent, directly regulated by their thermal environment (Farrell, 2016; Fry, 1947; Schulte *et al.*, 2015). The influence of temperature on the rate of physiological processes influencing metabolism leave fish vulnerable to changes in temperature but has also shaped ecological patterns and species distributions (Harada *et al.*, 2019). Ectotherms, like fishes, have an upper thermal tolerance limit at which biological processes breakdown, leading to reduced physiological performance and eventual mortality (Farrell, 2016; Kellermann *et al.*, 2012; Pörtner and Farrell, 2008; Schulte *et al.*, 2015). Evidence suggests that this threshold is likely related in part to the thermal history to which species have been exposed and are adapted (Comte and Olden, 2017; Eliason *et al.*, 2011; McKenzie *et al.*, 2021; Olsen *et al.*, 2021). Indeed, thermal performance has been shown to differ across salmon *Oncorhynchus* spp. populations of the same species (Abe *et al.*, 2019; Chen *et al.*, 2013; Eliason *et al.*, 2011; Eliason *et al.*, 2013; Stitt *et al.*, 2014; Whitney *et al.*, 2016), often matching the typical environmental temperatures to which they have encountered over prolonged periods. For example, Eliason *et al.* (2011) found that cardiorespiratory physiology varies among Fraser River sockeye salmon (*Oncorhynchus nerka*) populations and is related to the historical thermal conditions the species encountered during migration. In addition, on short timescales (hours to days to weeks), an individual fish can modify their physiology and morphology (i.e. acclimate) to compensate for a change in environmental temperature (Seebacher *et al.*, 2015a). Phenotypic plasticity is expected to play a key role in a population’s resilience to climate change (Seebacher *et al.*, 2015b); however, the capacity for phenotypic plasticity varies across species and populations (Sommer, 2020; Sparks *et al.*, 2017). Given this variation, biologists and managers cannot assume a single thermal threshold for a species (Zillig *et al.*, 2021). Instead, intraspecific comparisons are essential to ascertain the vulnerability of fish populations to climate change and to identify which populations may be more susceptible to extirpation and which may be more resilient to continued warming. These data can help managers adjust angling regulations and/or justify thermal reserves that are protective to native stocks.

Physiological thermal tolerance has been evaluated for many fish species (Beitinger and Bennett, 2000; [Bibr ref51][Bibr ref51]). The majority of this work has been done in a laboratory setting and perhaps the most common technique has focused on obtaining upper lethal temperature (CT-max, critical thermal maxima) with or without acclimation to particular temperatures (Beitinger and Bennett, 2000; Nati *et al.*, 2020). While CT-max provides a relevant lethal index to compare relative thermal tolerance and thermal safety margins (TSMs; TSM = CT-max − maximum environmental temperature) (Pinsky *et al.*, 2019; Sunday *et al.*, 2014; Vinagre *et al.*, 2019) across populations or species, it is not useful to define the functional thermal range for fitness-related performance (Farrell *et al.*, 2009; Rodnick *et al.*, 2004). Instead, aerobic scope (the difference between the standard and maximum oxygen consumption rate) is a measure of an organism’s aerobic capacity. While aerobic scope is commonly measured to understand optimal thermal conditions in ectotherms, including fish (Clark *et al.*, 2013; Farrell, 2016; Schulte *et al.*, 2015), the specific mechanisms that limit upper thermal tolerance among and across species is a subject of debate (see Jutfelt *et al.*, 2018, and associated references and commentary). For a given species, aerobic activities (e.g. locomotion, digestion, spawning and competition) at the organism level are feasible across a range of temperatures where aerobic scope is optimal. Beyond optimal temperatures (T_opt_), the fitness-related activities that allow an individual to thrive (e.g. locomotion, digestion) can become impaired. At a critical temperature threshold (T_crit_), aerobic scope is zero and mortality is imminent and inevitable (Whitney *et al.*, 2016). Functional warming tolerance (WT) calculated as the difference between the maximum environmental temperature and the upper temperature at which physiological performance ceases to be optimal to meet basic needs (T_pejus_) can be used to evaluate the functional vulnerability of populations to current temperatures and future warming. Incorporation of management and conservation approaches that consider both the physiological and ecological constraints and optimums of native fish species must be considered given evidence of climate regime shifts across the globe, the spatial and temporal variation and uncertainty in both the rate and extent of warming and uncertainty that exists in species responses in the near and long term.

In this study, we sought to evaluate how thermal performance varies in field acclimatized coastal cutthroat trout (*Oncorhynchus clarki clarki*) across four distinct watersheds in OR, USA, under acute warming scenarios, not unlike an extreme heat event. Specifically, we measured CT-max and oxygen consumption rates (for the calculation of aerobic scope) in trout at three ecologically relevant temperatures (ambient, current maximum stream temperature and a novel warm intended to reflect a climate change scenario) from four streams with variable hydrologic and thermal regimes (two watersheds with a warm thermal history; two watersheds with a cooler thermal history). All experiments were conducted stream side to mimic environmentally relevant conditions and minimize transport and handling stress, and any habituation effects from the laboratory residence. Thus, in contrast to a ‘common garden’ experiment (i.e. rearing individual fish from different populations in a common lab environment), this study design compared locally acclimatized fish, in their environmental conditions. We then used results of this study to calculate TSMs and functional WT to evaluate the degree to which coastal cutthroat trout may fare under current and future climate projections in stream temperature (~ +3°C; Jiménez Cisneros *et al.*, 2014). The implications of this study could challenge the use of a single thermal criterion applied to the same species across its geographic range providing both opportunities and challenges for how we think about managing thermal conditions under threat from development and climate change.

## Materials and Methods

Coastal cutthroat trout are widely distributed along the Western Pacific Coast, ranging from the Kenai Peninsula in Alaska to the Eel River in Northern California. They exhibit a diverse and flexible life history, residing in small headwater streams, higher gradient transition tributaries and large floodplain rivers (Trotter, 1989). This species exhibits both anadromous and stream-resident forms and vary considerably in body size, ranging from ~150 mm (mature stream resident) to over 500 mm (anadromous or sea-run). Because of this species’ expansive range and habitation of the entire river continuum (e.g. the longitudinal dimension of the stream ecosystem; Vannote *et al.*, 1980), they are an important species across which to compare thermal physiology.

### Study sites and fish sampling

**Figure 1:**
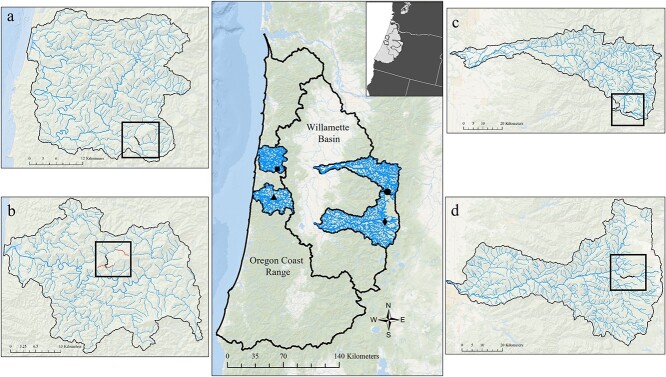
Reach locations (black line) within the Oregon Coast and Willamette basins where respirometry experiments occurred: (**a**) Little Rock Creek, Siletz (44.7206, −123.7117); (**b**) Fall Creek, Alsea (44.404, −123.7536); (**c**) N. Santiam River, N. Santiam (44.49841, −121.9837); and (**d**) White Branch Creek, McKenzie (44.1647, −122.0154). Fish were collected from within the reach locations, except in Fall Creek (panel b), where they were collected at locations indicated by red lines. Inset: Oregon positioned within the Northwestern USA.

Four streams from four distinct watersheds in OR, USA, were selected to conduct stream-side respirometry experiments (Fig. 1); two coastal watersheds, the Siletz and the Alsea, and two Willamette River Basin watersheds, the North Santiam (N. Santiam) and the McKenzie. We attempted to identify watersheds that had variable hydrologic regimes (defined by the magnitude of discharge and frequency, duration, timing and rate of change of flow events), which reflect interactions among many biophysical features of the ecosystem, environmental gradients and disturbance histories (Poff and Ward, 1990). We compared modelled water temperature data for each watershed using NorWeST (Isaak *et al.*, 2017) (Table 1). Specific stream-side respirometry sites within each of the four watersheds were chosen based on presence of coastal cutthroat trout, public ownership, ease of vehicle access, adequate space for equipment and low terrace heights along the creek to enable gravity pumping of stream water into tanks.

**Table 1:** Site characteristics including elevation, NorWeST estimates of maximum weekly maximum temperature (MWMT ± SE) for both baseline (mean 2002–2011) and 2080 projected values from streams where fish were collected and study temperature treatments (ambient, maximum and climate treatments).

Temperature ^0^ C								
Location	Stream	Elevation (m)	MWMT^a^ baseline	MWMT^a^ 2080	Ambient min–max	Ambient mean (SD)	Max treatment	Climate treatment
Alsea	Fall Creek Tributary	109	16.97 ± 2.53	18.77	16.6–20.0	18.4 (1.17)	19	22
Siletz	Little Rock Creek	72	18.87 ± 2.64	20.79	16.6–20.0	18.8 (0.71)	19	22
McKenzie	White Branch Creek	642	15.60 ± 2.62	17.32	10.4–13.4	11.8 (0.90)	14	19
N. Santiam	N. Santiam River	1127	15.79 ± 2.35	17.52	6.3–10.3	8.1 (1.31)	14	19

The respirometry experiments in Alsea, McKenzie and N. Santiam were conducted in August 2018 and in Siletz in August 2019. The Ambient temperatures for the Alsea were from Fall Creek where the experiment occurred.

^a^ Isaak *et al.*, 2017.

The streams selected in the coastal watersheds (Fig. 1) are positioned in the Western Oregon Coast Range Mountains (Alsea and Siletz) at elevations below 500 m. Both streams are underlain by marine sedimentary geology, primarily sandstone and siltstone of the Tyee formation and are characterized by primarily coniferous forest and secondarily deciduous cover with shrub-scrub understory. Coastal Oregon is characterized by wet winters and dry summers with mild temperatures year-round. These systems exhibit rain-driven hydrologic regimes that are reactive and have little storage capacity. Annual precipitation ranges between 165 and 228 cm. Stream temperatures during the study (2018–2019) ranged from 16°C to 20°C and from 17°C to 20°C for Alsea and Siletz, respectively (Table 1). Coastal cutthroat trout were collected from tributaries of Fall Creek in the Alsea watershed and Little Rock Creek in the Siletz (Fig. 1a and b).

The streams located in watersheds along the western slopes of the Cascade Range in the Willamette River basin (McKenzie and N. Santiam) (Fig. 1) are both positioned above 500 m. Both streams are primarily underlain by porous volcanic geologies, allowing snow and rain runoff to filter and flow far beneath the surface. Both streams were at elevations in the rain–snow transition zones. While the McKenzie River and many of its tributaries (including the study stream) are spring fed, helping to maintain flow and constant seasonal stream temperatures, the aquifers in the upper reaches of the N. Santiam River (which includes the study stream) are variable in supply (Synder *et al.*, 2002). Both watersheds are characterized by steep forested uplands and alluvial lowlands and include a number of impoundments (dams) lower in the watersheds. Stream temperatures during the study ranged from 10°C to 13°C and from 6°C to 10°C for McKenzie and N. Santiam, respectively (Table 1). Coastal cutthroat trout were collected from White Branch Creek in the McKenzie and the upper reaches of the N. Santiam River in the N. Santiam (Fig. 1c and d).

### Acclimation temperature treatments

For all watersheds, coastal cutthroat trout [target size range, 90–150 mm fork length (FL)] were collected by electrofishing each day in the late morning or early afternoon during August and were allowed several hours to recover from capture and to adjust to the holding tank. Fish were then acclimated overnight to their treatment temperature. Eight fish were evaluated per temperature treatment, with a total of 24 fish caught per watershed throughout the duration of the experiment. The fish were placed in a 100-L fibreglass holding tank positioned immediately adjacent to the stream. Fresh water was circulated through the tank in a flow-through system using a water pump. Three large airstones were placed in the tank to ensure dissolved oxygen levels were maintained at >90% air saturation (Fig. S2). Water temperature was monitored hourly and maintained at the treatment temperature. Electrical power to run all experimental equipment was supplied by two generators (EU7000IS and EU3000IS; Honda Motor Company Ltd, Japan). All procedures were approved by University of California, Santa Barbara Institutional Animal Care and Use Committee.

The temperature treatments were selected to reflect the specific thermal conditions and to facilitate comparisons across watersheds (Table 1). The first temperature treatment reflected the ambient water temperature with the natural diurnal fluctuation (‘Ambient’ treatment). The second temperature treatment was set at the maximum summer temperature observed in the days preceding the experiment (‘Max’ treatment). The third temperature treatment was set 3°C higher than the maximum temperature and represented the novel warm conditions fish will likely have to endure to persist into the future (from here on named the ‘Climate’ treatment) (Table 1). Each acclimation period was ~18 h in duration, which was selected as ecologically relevant and representative of an acute warming event. Although full acclimation processes are anticipated to take >18 h to occur, studies have shown that metabolism can acclimate rapidly, within 1–2 days in fish species [goldfish (*Carassius auratus*): Klicka, 1965; minnows (*Umbra limi*): Hanson and Stanley, 1970; curimbatá (*Prochilodus scrofa*): Barrionuevo and Fernandes, 1998]. While the acclimation duration used in this study was relatively short, our interest was to evaluate the thermal sensitivity and the capacity for rapid phenotypic plasticity of these fish to an acute thermal warming event similar to a potentially abrupt but brief environmental temperature shift, rather than to evaluate the full acclimation capacity of fish to chronic, extreme thermal conditions. Given that cutthroat trout live in thermally variable environments, it is ecologically relevant to understand how they can rapidly respond to changing temperatures.

For the Ambient treatment, the water temperature in the holding tank was not adjusted or heated in any way, reflecting the natural diurnal fluctuation in the stream. For the Max and Climate treatments, the water temperature of the holding tank was increased from ambient to the test temperature (by ~1°C per hour) via an adjacent header tank that was equipped with a submerged water heater (Smart One Easy Plug Axial Heaters) powered by a generator (Fig. S2). Fish were not fed to reduce the potential that digestion processes affecting metabolism would confound the respirometry measurements.

Temperature profiles during the ambient respirometry trial (early to late August for all watersheds) were distinctly different between the coastal watersheds and Willamette basin watersheds (Table 1; Fig. S1). The two coastal watershed streams were notably warmer than those in the Willamette basin watersheds. The pattern of diurnal variation in temperature was similar across all four streams and resulted in a 2.4–3.9°C change for a 24-h period. The peak temperature for all streams occurred between 3:30 pm and 6 pm in the afternoon/evening and decreased soon thereafter (Fig. S1).

### Field respirometry

An eight-chamber intermittent flow-through respirometry system was constructed to measure oxygen consumption (Fig. S2). The system consists of eight clear plastic 2-L chambers that were approximately 80× the volume of the fish. Each chamber was equipped with two water pumps. A small Sicce Micra pump (~5 L/min) flushed fresh water through the chamber and the Eheim compact pump (~5 L/min) recirculated water continuously past a fibre-optic oxygen probe (robust Firesting O_2_ probe) that monitored oxygen levels in the chambers. Each oxygen probe was attached to an external optical oxygen meter (4-channel FireSting O_2_, PyroScience, Germany) that continuously measured dissolved oxygen levels in the respirometry chamber that were later used to calculate oxygen consumption rates. Each 4-channel FireSting system was also equipped with a fibre-optic temperature probe to compensate oxygen measurements given varying stream temperatures. Two chambers were positioned in a 102-L tank (eight chambers, four tanks). Water temperature was measured continuously in two of the four tanks containing the submerged respirometry chambers. We assumed temperatures were similar in the other tanks but placed digital thermometers (resolution 0.1°C; accuracy +/− 1°C) in all tanks to ensure that temperatures stayed relatively similar in all the tanks throughout the experiment. The digital thermometers were on average 0.63 cooler than the fibre-optic temperature probes. The tanks received either ambient water from the proximate stream via a water pump or heated water from an adjacent header tank that was equipped with 1–2 SmartOne Easy Plug Axial Heaters (1700 watts, 120 volts). Two large airstones were placed in the header tank to ensure dissolved oxygen levels were maintained at >90% saturation in the chambers.

**Figure 2:**
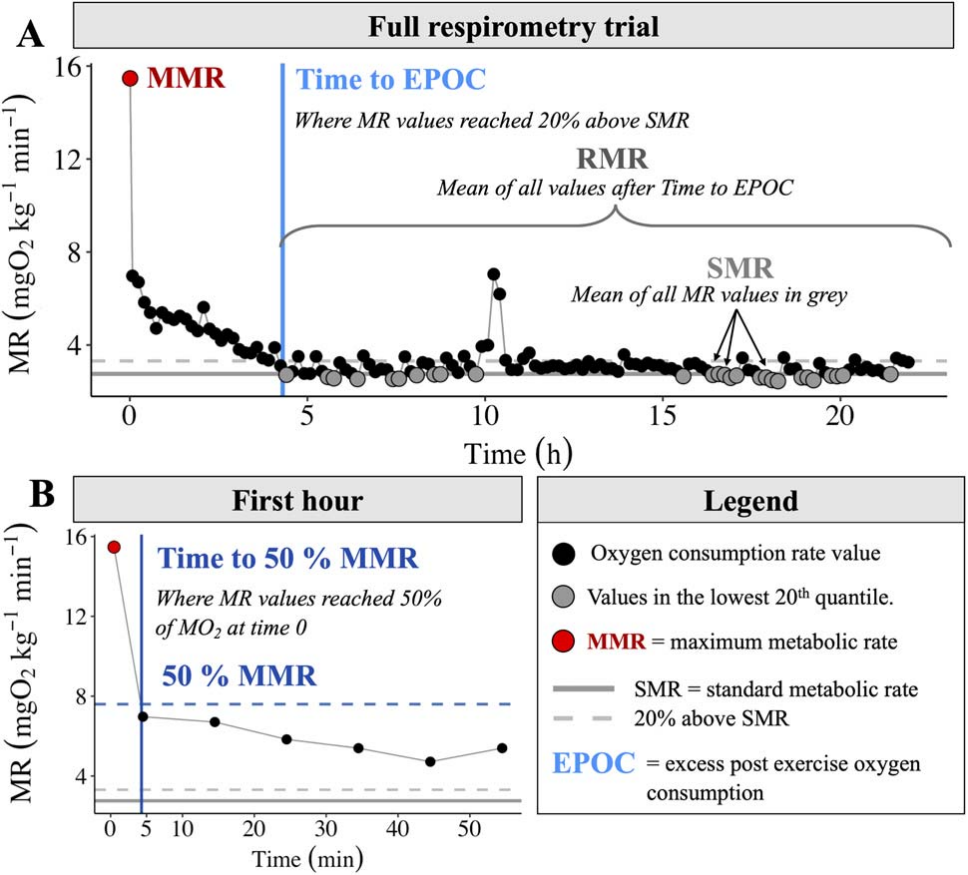
Representative example of a trace plot and calculations. (**A**) A full overnight respirometry trial showing all recorded oxygen consumption rates; the MMR was the highest metabolic rate measurement recorded. This trend was used to find time to full recovery or time to EPOC. To find where metabolic rate returned to 20% above SMR level, a smooth fit function was used and the intersection of 20% SMR and this smoothed line was defined as time to EPOC. (**B**) The time to 50% MMR was found using the same method as the time to EPOC. Time 0 and therefore MMR was the time of the first recorded measurement.

Individual fish were manually chased by hand for 3 min to exhaustion in a large cooler (45.4 L). This is a standard protocol that has been frequently used to exhaust fish (Norin and Clark, 2016; Little *et al.*, 2020). The fish were then air-exposed for 30 s before being immediately placed in a respirometry chamber at which time measurement of oxygen consumption began and continued for 18–24 h (Eliason *et al.*, 2008) (Fig. 2). The system operated as a closed respirometer during short measurement phases (6 min) when the chamber is closed, but between measurement phases, water in the respirometer is flushed (for 4 min) and replaced to prevent hypoxia and the build-up of metabolites (10 min measurement:flush cycles, resulting in 108–144 total measurements per trial). After 18–24 h, the fish were removed from the chambers and weighed (body mass in grammes) and measured (FL, in mm). The fish were then returned to the holding tank at their treatment temperature. If the treatment temperature was anything other than ambient, the temperature of the holding tank was slowly reduced to the ambient stream temperature. Before the first experiment (e.g. Ambient treatment) and following the last experiment (e.g. Climate treatment), oxygen consumption was measured in an empty respirometer (e.g. no fish) to measure background oxygen content (e.g. respiration). Background respiration was low across watersheds (0.15–2.1% of fish respiration).

### Critical thermal maximum

Approximately 1 h after the respirometry experiment for the ambient treatment, the same fish underwent a standard critical thermal maximum (CT-max) protocol (Fangue *et al.*, 2006). Fish were placed in a large (45.4 L) cooler with aerated fresh water. Water temperature was increased by 0.3°C per minute using a stainless-steel immersion heater coil attached to a water pump. Heating rates during the CT-max test were similar across watersheds. While we strived to heat the water 1°C every 3 min, there was some variability. The average heating rate for all locations was 0.96–0.99°C and ranged from a minimum heating rate of 0.5–0.7°C to a maximum heating rate of 1.3–1.5°C, every 3 min. When an individual fish lost equilibrium, it was immediately removed and the time and temperature was recorded. The fish was then placed in a recovery tank that was ~5 C cooler than the CT-max temperature and the water temperature was gradually reduced back to ambient. At the conclusion of the CT-max test, the fish were allowed several hours to recover at ambient water temperatures before being returned to the stream.

### Data and statistical analysis

We followed methods outlined in Rosewarne *et al.* (2016) to calculate fish oxygen consumption rate (*Ṁ*O_2_ mg O_2_ kg^−1^ min^−1^; equation 1), the final calculations yielding one *Ṁ*O_2_ value per fish per measurement phase (6 min measurement phase, 4 min flush) (Fig. 2). We corrected each measurement for background respiration that may have been caused by micro-organisms residing in the tubing or chambers:

*Ṁ*O_2_ = [Δ [O_2_] _fish_ * (V_chamber_—Mass)]—[Δ [O_2_] _background_ * V_chamber_] × Mass^−1^, (Equation 1)

where *Ṁ*O_2_ is the oxygen consumption rate for each measurement phase, Δ [O_2_]_fish_ is the rate of decrease in oxygen content in the respirometer over the course of measurement (mg O_2_ L^−1^ min^−1^), Δ [O_2_] _background_ is the rate of decrease in oxygen content in the empty respirometer over the course of measurement (mg O_2_ L^−1^ min^−1^), V_chamber_ is the volume of the respirometer (L) and Mass is the body mass of the individual fish (kg). Because metabolism often scales allometrically with body size in fish (Jerde *et al.*, 2019), we checked for body mass effects on oxygen consumption rates (MMR and SMR) in our study. We evaluated linear regression between natural log transformed raw oxygen consumption rates (*ln* mg O_2_ L^−1^ min^−1^) and natural log transformed body mass of each individual (*ln* kg) and used the regression values to confirm that allometric mass correction on our measurements was not necessary (Fig. S4; Table S9). Lastly, Fulton’s body condition factor was measured as:

K = 100 000 M/L^3^,

where *M* is the mass of the fish in grammes and *L* is the fork length of the fish in millimetres. Differences between body morphometrics (mass and length) and condition factor were analysed using an Analysis of Variance test (ANOVA).

### SMR, MMR and aerobic scope

We evaluated several metrics to understand the extent to which oxygen consumption rate differs among individuals and across different watersheds. Standard metabolic rate (SMR; mg O_2_ kg^−1^ min^−1^) is described as the minimum maintenance metabolism or basic cost of living (i.e. fish in a resting, non-reproductive, post-absorptive state). To calculate SMR, we extracted the lowest 20% of all recorded MO_2_ values (*N* = 32–274) and calculated the mean value for each fish (SEM = 115.2 ± 4.3) (Chabot and McKenzie, 2016) (Fig. 2). For the Ambient treatment, SMR was calculated from all recorded MO_2_ values, even though temperature fluctuated throughout the experiment (Fig. S1). Notably, following recovery from the chase [i.e. after excess post-exercise oxygen consumption (EPOC) was complete], MO_2_ remained remarkably constant for an individual fish even though temperature fluctuated (Fig. S5). The corresponding temperature values for each MO_2_ measurement used in the SMR calculation were averaged to determine the mean temperature for SMR for each fish. Maximum metabolic rate (MMR; mg O_2_ kg^−1^ min^−1^), the upper boundary for aerobic metabolism that is achievable by an animal, was measured immediately after the 3-min chase and 30-s air exposure once fish were placed in the respirometry chambers (Little *et al.*, 2020). For the ambient trial, the temperature at which MMR was measured did not match the SMR temperature because SMR was measured over a range of temperatures during the diurnal cycle. For the ambient treatments, the chase and air exposure occurred at approximately the same time during the day (between 9:00 and 10:00 am) for each watershed, with chase temperatures averaging 16.5 C, 18.1 C, 10.5 C and 7.4 C for the Alsea, Siletz, McKenzie and N. Santiam, respectively. Absolute aerobic scope (AAS = MMR − SMR; mg O_2_ kg^−1^ min^−1^), represents the absolute energy available to thrive in the environment (e.g. move, find food, migrate, etc.) and factorial aerobic scope (FAS = MMR/SMR) was calculated to understand whether a metabolic constraint might arise as temperatures increase (Halsey *et al.*, 2018).

### Recovery performance

We evaluated recovery performance for each individual by calculating the time it took for fish to recover to 50% of their MMR (Time [MMR_50_]) (Kraskura *et al.*, 2021). This is an indication of short-term recovery from exhaustive exercise and is an estimate for the amount of recovery time necessary before the fish can resume normal activities (e.g. swimming, foraging, etc.). Salmonids have an exceptional ability to recover, which has been demonstrated by their repeat swim performance with only 45 min of break between the consecutive swim tests (e.g. Eliason *et al.*, 2013; Farrell *et al.*, 2003; Farrell *et al.*, 1998). In these studies fish decreased their oxygen consumption to 30–70% of MMR, therefore the chosen time to recover to 50% MMR is an ecologically and physiologically relevant recovery metric (Kraskura *et al.*, 2021). Full recovery was determined as the time (h) to end of EPOC, which in this study was when MO_2_ values reached 20% above the SMR value. To calculate these metrics we fit a smoothed curve over all MO_2_ values (smooth.spline R); the time to EPOC was the time when the smoothed line intersected the 20% above SMR threshold (Fig. 2). To evaluate differences in SMR, MMR, AAS, FAS and Time [MMR_50_] estimates within each of the four watersheds, we used the non-parametric Kruskal–Wallis test. Where significant differences occurred (*P* < 0.05), the *post hoc* Dunn’s Multiple Comparison test was used to identify which watersheds differed.

### Routine metabolic rate and Q10

Routine metabolic rate (RMR, mg O_2_ kg^−1^ min^−1^) was evaluated using MO_2_ values [*N* = 3–274, range; 121.0 ± 4.1 measurements, mean (SEM)] measured after the fish had fully recovered from the exhaustive chase (i.e. after EPOC was complete). Due to the diurnal temperature fluctuations during the Ambient temperature treatment, we obtained RMR measurements for fish at 4, 3, 3 and 5 different temperatures for the Alsea, Siletz, McKenzie, and N. Santiam watersheds, respectively. To be included in the RMR estimates for a given temperature, individual fish were required to have at least 3 RMR measurements at that temperature. This allowed for an examination of metabolic response at a range of temperatures experienced by individual fish. To evaluate differences in RMR values within each watershed, we developed a log-normal linear mixed model fit with RMR as the response variable and two independent fixed-effect variables: (i) temperature during the measurement (continuous variable) and (ii) basin (Alsea, McKenzie, Siletz and N. Santiam). The random intercept effects were treatment (Climate, Max and Ambient) and individual fish (lme4 package in R; Bates *et al.*, 2015). Treatment was added as a random intercept effect to account for non-independence in these temperature treatments across basins and individual fish were included as a random effect to account for non-independence between data points across temperatures. Q10 values, which provide insight into the degree to which routine and SMRs are influenced by temperature, were calculated for all locations using following equation:

$$ Q10={\left(\frac{R2}{R1}\right)}^{\frac{10}{T2-T1}}, $$

where *R2 =* MO_2_ at Climate treatment temperature, *R1 =* MO_2_ at lowest Ambient temperature, *T2 =* temperature at *R2* and *T1* = temperature at *R1*. A Q10 value greater than 1 indicates that rates are increasing with temperature, less than 1 indicates that rates are decreasing with temperature and a value equal to 1 indicates that the rate is temperature independent.

For each of the watersheds, one temperature treatment, 19°C, was shared. For the Coastal watersheds this represented the Max temperature treatment; in the Willamette basin watersheds, 19°C represented the Climate temperature treatment. Using the same analytical approach (Kruskal–Wallis test, Dunn’s test), we also evaluated whether SMR, MMR, AAS, FAS and Time [MMR_50_] estimates varied at 19°C across the four watersheds. All analyses were conducted using R studio version 1.0.143. Significance level for all statistical tests was α = 0.05.

### Evaluating climate implications

Metabolic rate is a temperature-dependent performance trait that can influence species distributions and vulnerability (Schulte *et al.*, 2011). When trying to understand species response to a changing climate, considering the interaction between exposure and thermal limits is important. We calculated two measures to understand species resiliencies to increasing stream temperatures. First, TSM was calculated as the difference between an individual’s CT-max and the maximum temperature of the habitat (environmental temperature). This measure can provide an approximation of the amount of environmental warming an organism can tolerate before death is imminent. As TSM values get smaller, there is less capacity to deal with warming (or higher probability of death) (Pinsky *et al.*, 2019; Sunday *et al.*, 2014; Vinagre *et al.*, 2019; Walters *et al.*, 2018).

Second, we calculated functional WT. WT was calculated as the difference between an organism’s T_pejus_ and the maximum temperature of that organism’s habitat. The definition for T_pejus_ is expected vary across species and even the life cycle, as the physiological requirements to thrive differ. We define T_pejus_ for coastal cutthroat trout as the temperature at which an individual’s FAS is equal to 3 (Eliason *et al.*, 2022). We specifically chose an FAS threshold rather than an AAS threshold because FAS can indicate when a metabolic constraint is developing: the metabolic floor is a much greater concern than the metabolic ceiling in this system (Eliason *et al.*, 2022). Cutthroat trout at this life stage are highly unlikely to routinely use MMR and require their full, maximal AAS. Thus, setting T_pejus_ as a high AAS threshold has little ecological relevance in this system (Farrell, 2016). In contrast, FAS can help us infer how much of the energetic capacity is being allocated to simple maintenance costs. As temperatures increase, SMR costs increase, and accordingly a greater proportion of the energy intake must be allocated simply to support maintenance metabolism. Any energy allocated to SMR is not being allocated to other critical activities such as growth or reproduction. Higher maintenance costs may require the fish to spend more time foraging and eating to support the increased metabolic demand and also have sufficient energy to meet other activities (e.g. growth) requirements, which may confer lost opportunity costs (territory, mates) and increase susceptibility to predation. In addition, in order to thrive, cutthroat trout must have the energetic capacity to digest a meal. Rainbow trout double their metabolism during digestion of a moderate sized meal (2% of body mass, Eliason *et al.*, 2008), thus an FAS of 2 is necessary for digestion (Farrell, 2016). However, to thrive, a fish must also be able to digest a meal and have sufficient scope remaining for other activities (e.g. swimming, defending territory, remaining vigilant, growth, reproduction; Jutfelt *et al.*, 2021). Thus, we propose an FAS threshold of 3 for a subadult salmonid to be able to thrive (Eliason *et al.*, 2022). For a different species and life stage, different criteria will be appropriate. For example, for migrating adult sockeye salmon populations maximally swimming hundreds of kilometres up the Fraser River, BC, Canada, to reach distant spawning grounds are expected to have a T_pejus_ threshold of 90% of AAS (Eliason *et al.*, 2011). All told, the WT measure can provide an approximation of the amount of environmental warming an organism can tolerate before performance declines. Small WT values indicate that the species is living close to their thermal edge and small amounts of warming will likely result in decreases in performance.

**Figure 3:**
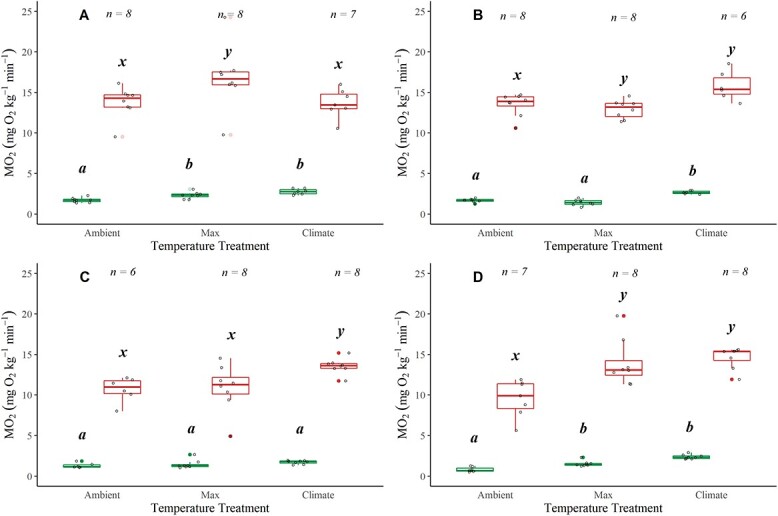
Boxplots displaying SMRs and MMRs plotted at mean ambient temperature, maximum and climate treatments for Alsea (**A**), Siletz (**B**), McKenzie (**C**) and N. Santiam (**D**) watersheds. Boxplots show distribution within the 25th and 75th percentiles, the median (centre line) and the 95% confidence intervals. Open dots represent individual fish and have been jittered around boxplots. Sample size denoted at the top of each plot. Significant pairwise differences (Dunn’s *post hoc* test; *P* < 0.05) in means within a location are noted by lower-case grouping letters (a, b and c for SMR; x, y and z for MMR). Different letters indicate significant pairwise differences.

To calculate T_pejus_, we developed linear models between FAS and average study temperatures for each watershed (Table S3). We then used the equation to predict the temperature at which FAS is estimated to be ~3. When calculating both TSM and WT, we used the maximum weekly maximum temperature (MWMT) estimates available from NorWeST (Isaak *et al.*, 2017) to represent the maximum environmental temperature. Kruskal–Wallis tests were used to evaluate whether the distributions of both WT and TSM were statistically different among watersheds. Where differences were detected for either WT or TSM, the *post hoc* Dunn’s Multiple Comparison test was used to identify differences between watersheds.

**Figure 4:**
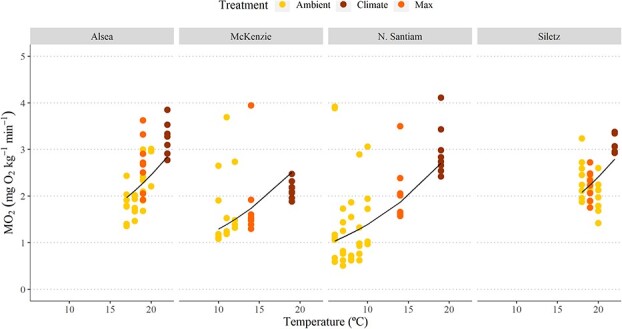
A maximum likelihood mixed model was used to describe how RMR depends on temperature in coastal cutthroat trout. The fitted equation is ln(RMR) ~ 1.076 (Temperature) + $\square$ (unique to watershed) + error. Coefficients ($\square$) for each watershed were as follows: Alsea, −0.5749; McKenzie, −0.4784; N. Santiam, −0.4042; and Siletz, −0.5926. Treatment and individual fish unique to each watershed were non-independent factors. Treatment has positive effect on these slopes with Climate > Max > Ambient. See Table S2 for model output.

## Results

Across all locations and treatments mean individual body mass ranged from 17.5 to 29.8 g (22.8 ± 1.00 g; mean ± SEM), individual fork lengths ranged from 100 to 181 mm (130.2 ± 1.92 mm; mean ± SEM) and condition factor ranged from 0.93 to 1.13 (1.00 ± 0.02; mean ± SEM) (Fig. S4; Table S1). Body mass, length and condition factor did not differ across temperature treatments within a watershed, or across watersheds (ANOVA, *P* > 0.05; Fig. S4; Table S1). All coastal cutthroat trout in the Willamette (McKenzie, N. Santiam) are resident fish, but the coastal trout (Alsea, Siletz) could be a mix of sea-run and resident fish.

**Figure 5:**
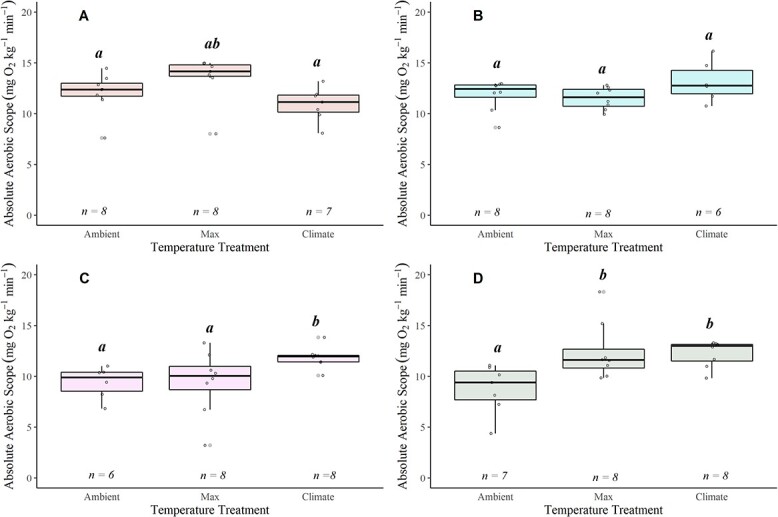
Boxplots displaying absolute aerobic scope (AAS = MMR − SMR) for coastal cutthroat in the Alsea (**A**), Siletz (**B**), McKenzie (**C**) and N. Santiam (**D**) watersheds. The lower and upper boundaries of the boxplot represent the interquartile range (25th and 75th percentiles), the darker centre line delineates the median and the whiskers indicate the minimum and maximum. Points represent individual fish. Significant pairwise differences (Dunn’s *post hoc* test; *P* < 0.05) in means within a location are noted by lower-case grouping letters. Different letters indicate significant pairwise differences.

### Critical thermal maxima

The Siletz watershed had the highest mean CT-max (30.375°C SE 0.059) followed by the Alsea (Table S2). The mean CT-max for the coastal watersheds were significantly higher (by over 2.5°C) compared with the sample of fish from the Willamette basin watersheds (Table S2). Of note specifically in the McKenzie watershed, two fish lost equilibrium ~10 min before the rest of the fish. The remaining fish lost equilibrium within 40 s of each other, which was similar to the results seen in the other watersheds, with a minimum of 1 s to maximum of 44 s.

### SMR and MMR

SMRs increased with an increase in temperature for coastal cutthroat trout from all watersheds (Fig. 3; Table S5): Alsea ($\chi$^2^ = 14.591, *P* < 0.001), Siletz ($\chi$^2^ = 14.04, *P* < 0.001), McKenzie ($\chi$^2^ = 6.952, *P* = 0.030) and N. Santiam ($\chi$^2^ = 17.615, *P* < 0.001). In all watersheds the SMR in the Climate treatment was significantly higher than in the Ambient treatments (Dunn’ *post hoc P* < 0.05), and in all watersheds except the Alsea, the SMR during the Climate treatment was significantly higher than the Max treatment (Dunn’s *post hoc P* < 0.05; Fig. 3A; Table S5). The coastal cutthroat trout in the N. Santiam watershed had the lowest ambient SMR values, which were also associated with the lowest water temperatures observed across the four watersheds (Fig. 3D; Table S5).

MMRs increased with an increase in temperatures though the specific pattern varied across watersheds (Fig. 3). MMR values were statistically different across treatments in all watersheds: Alsea ($\chi$^2^ = 7.417, *P* = 0.024), Siletz ($\chi$^2^ = 9.690, *P* = 0.007), McKenzie ($\chi$^2^ = 8.757, *P* = 0.012) and N. Santiam ($\chi$^2^ = 12.99, *P* = 0.001). In the Alsea, there was a significant decrease in MMR during the Climate trial (Dunn’s *post hoc P* < 0.05; Fig. 3A; Table S5). Q10 values (comparing Ambient with Climate temperatures) for each location were 3.25, 2.02, 1.17 and 2.01 for Alsea, Siletz, McKenzie and N. Santiam, respectively, indicating that there was variable temperature sensitivity across watersheds.

At the shared 19°C temperature, the Alsea and the N. Santiam coastal cutthroat trout had higher SMR than the Siletz and the McKenzie ($\chi$^2^ = 20.645, *P* < 0.001) (Fig. 7A; Table S7). The MMR values were significantly higher in the Alsea compared with the fish in the other watersheds ($\chi$^2^ = 13.636, *P* = 0.003) (Fig. 7B; Table S7).

### Aerobic scope

There were three distinct patterns for AAS with temperature: both the N. Santiam and McKenzie watersheds showed a significant increase in AAS with an increase in temperature (respectively, $\chi$^2^ = 9.642, *P* = 0.008,$\chi$^2^ = 6.952, *P* = 0.031) (Table S6; Fig. 5C and D); the Alsea showed a significant decrease in AAS at 22°C, the climate treatment ($\chi$^2^ = 8.834, *P* = 0.012) (Table S6; Fig. 5A); and the Siletz displayed no change in AAS with temperature (Table S6; Fig. 5B).

**Figure 6:**
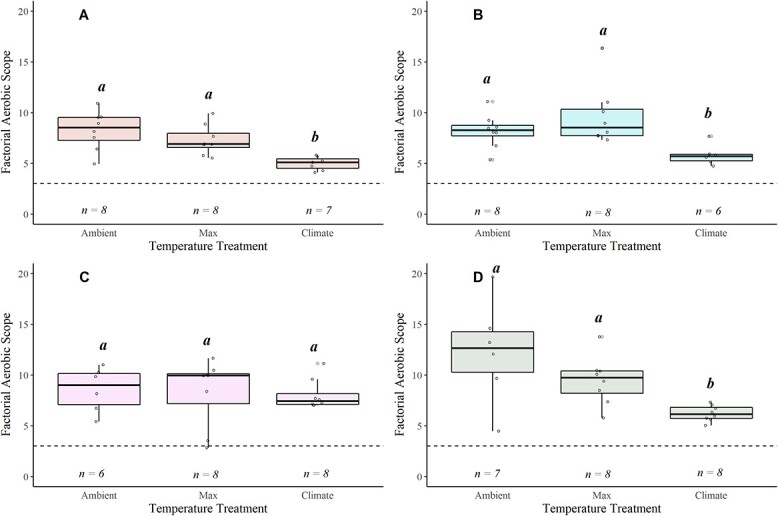
Boxplots displaying factorial aerobic scope (FAS = MMR/SMR) for coastal cutthroat in the Alsea (A), Siletz (B), McKenzie (C) and N. Santiam (D) watersheds. The lower and upper boundaries of the boxplot represent the interquartile range (25th and 75th percentiles), the darker centre line delineates the median and the whiskers indicate the minimum and maximum. The dashed horizontal line indicates the estimated FAS needed to thrive for this life stage (i.e. FAS = 3). Significant pairwise differences (Dunn’s *post hoc* test; *P* < 0.05) in means within a location are noted by lower-case grouping letters. Different letters indicate significant pairwise differences.

**Figure 7:**
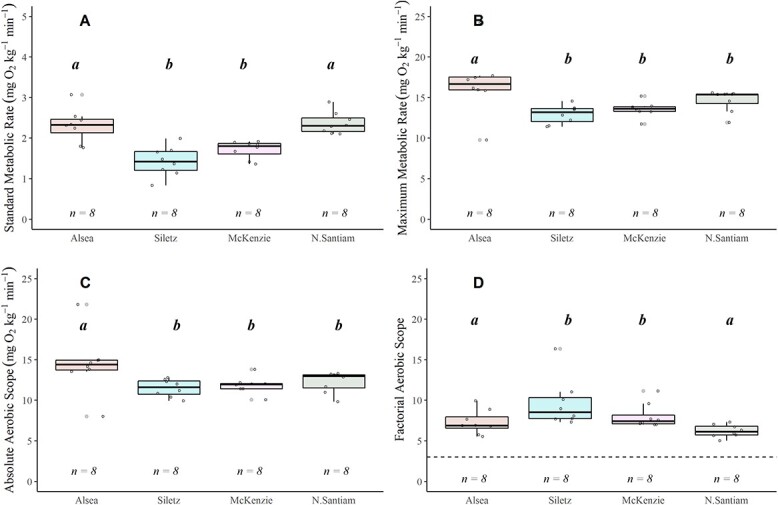
Boxplots displaying SMR (A), MMR (B), AAS (C) and FAS (D) for coastal cutthroat trout at the shared temperature of 19°C. The lower and upper boundaries of the boxplot represent the interquartile range (25th and 75th percentiles), the darker centre line delineates the median and the whiskers indicate the minimum and maximum. The dashed horizontal line indicates the estimated FAS needed to thrive for this life stage (i.e. FAS = 3). Significant pairwise differences (Dunn’s *post hoc* test; *P* < 0.05) in means within a location are noted by lower-case grouping letters. Different letters indicate significant pairwise differences

FAS decreased with increasing water temperature in most of the watersheds: Alsea ($\chi$^2^ = 11.209, *P* = 0.003), Siletz ($\chi$^2^ = 9.966, *P* = 0.006) and N. Santiam ($\chi$^2^ = 10.052, *P* = 0.006). In contrast, FAS was unaffected by temperature in the McKenzie watershed (Fig. 6; Table S6). The N. Santiam watershed had the most precipitous decline (48%) in FAS across temperature (Mean ± SEM): Ambient = 12.025 ± 2.250, Max = 9.462 ± 0.841, Climate = 6.224 ± 0.274), (Fig. 6D). When comparing at 19°C, AAS and FAS differed across watersheds (Fig. 7C and D, Table S6). Specifically, AAS was significantly higher in the Alsea compared with the Siletz ($\chi$^2^ = 10.432, *P* = 0.015; Table S7; Fig. 7C), while FAS differed between the Siletz and N. Santiam ($\chi$^2^ = 16.102, *P* = 0.001; Table S7; Fig. 7D).

### Recovery

**Figure 8:**
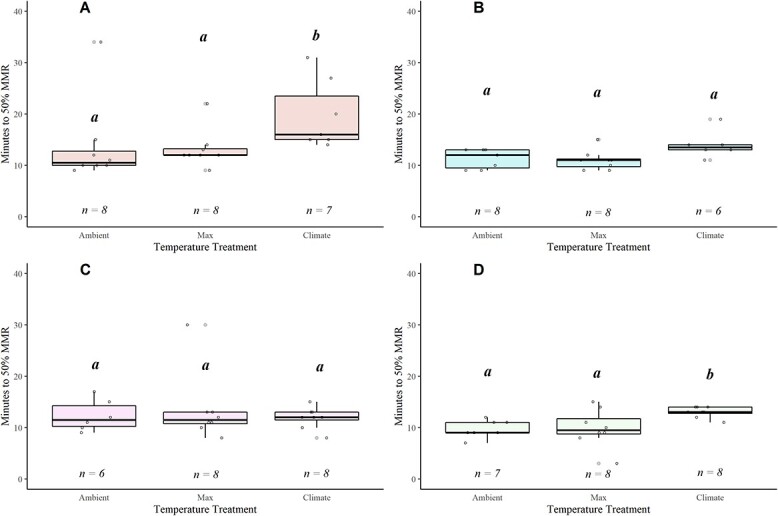
Boxplot displaying minutes to recover to 50% of the MMR across all treatment trials for Alsea (A), Siletz (B), McKenzie (C) and N. Santiam (D). The lower and upper boundaries of the boxplot represent the interquartile range (25th and 75th percentiles), the darker centre line delineates the median and the whiskers indicate the minimum and maximum. Open dots represent individual fish and have been jittered around boxplots. Sample size denoted at the bottom of each plot. Significant pairwise differences (Dunn’s *post hoc* test; *P* < 0.05) in means within a location are noted by lower-case grouping letters. Different letters indicate significant pairwise differences. Number of fish per treatment also referenced (*n*)

Overall, the fish recovered rapidly from the exhaustive chase protocol; mean Time [MMR50] ranged from 10 to 24 min (Fig. 8; Table S8). Recovery time increased significantly with warming for fish in two of the watersheds (Alsea and N. Santiam; Fig. 8A and D; Table S8). In contrast, the McKenzie and Siletz fish maintained a stable recovery duration across all temperatures (Fig. 8B and C; Table S8). When comparing across watersheds at the shared 19°C temperature, there were no differences in Time[MMR_50_] (Table S8).

### Routine metabolic rates

RMRs for coastal cutthroat showed similar patterns across all watersheds, increasing exponentially with an increase in water temperature (Fig. 4, Table S4). Q10 values (comparing lowest Ambient and Climate temperature treatments) for each watershed were 3.20, 1.93, 1.48 and 1.48 for Alsea (17–22°C), Siletz (18–22°C), McKenzie (10–19°C) and N. Santiam watersheds (6–19°C), respectively.

### Climate implications: Functional WT and TSM

**Figure 9:**
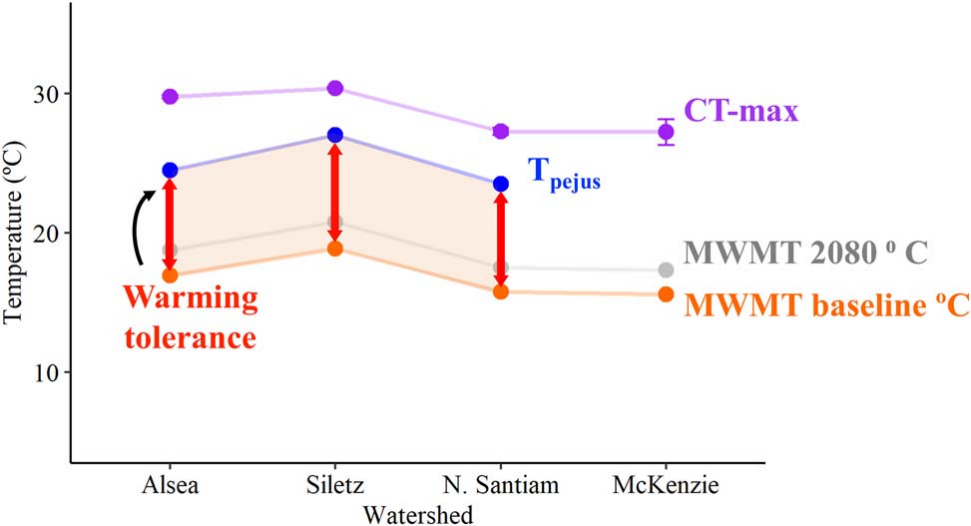
Displayed for each watershed, the mean critical thermal maximum (purple), the temperature at which Tpejus occurs as modelled (blue; see Table S3), baseline modelled maximum weekly maximum temperatures (NorWeST) from the streams where fish were collected (orange; Isaak *et al.*, 2017), future (2080) projected maximum weekly maximum temperatures (NorWeST) from the streams where fish were collected (grey; Isaak *et al.*, 2017) and WT values (red; see Table S2).

WT values were similar across the watersheds. The N. Santiam and Alsea had similar WT values though the underlying T_pejus_ and baseline MWMT varied (Fig. 9; Table S2). We were unable to calculate WT for the fish in the McKenzie watershed given the stability of FAS across temperatures and the inability to define T_pejus_. Fish from the Alsea had the highest mean TSM (12.805 ± 0.0115°C) followed by the McKenzie (11.652 ± 0.938°C) where there was the greatest variability between individuals, which makes sense given the variation in CT-max (Mckenzie CT-max range, 22.6–29.4) (Table S2). The N. Santiam and Siletz had the same mean TSM (11.5°C) though the fundamental values differed; the Siletz had the highest CT-max and a baseline MWMT that was nearly 3 degrees higher than the N. Santiam. The N. Santiam had the lowest CT-max and one of the lowest baseline MWMT (Fig. 9; Table 1). Across all watersheds, future 2080 projections of maximum weekly maximum temperatures resulted in lower WT and TSM values (Fig. 9; Table S2).

## Discussion

In this study, we discovered considerable intraspecific variation in physiological performance and thermal tolerance across coastal cutthroat trout from four distinct watersheds in Oregon. While the diurnal variability was similar across the watersheds (range, 2.4 C in Siletz to 3.9 C in the N. Santiam), the extent of warming (e.g. min, max) did differ. Fish from the cooler, more stable, spring-fed system (McKenzie) had the most variable CT-max values while also having the most stable FAS values across all temperature treatments. Fish from the warmest thermal regimes (e.g. Siletz) had higher CT-max values and had less variability in AAS. Conversely, fish from cooler thermal regimes exposed to novel warm conditions (e.g. Alsea and N. Santiam) had the only decrease in MMR during the Climate trial (Alsea) and the most precipitous decline in FAS (N. Santiam). Thermal history appeared to be more descriptive of the thermal metabolic response than watershed. However, fish from all watersheds appear to be at low risk of acute thermal stress because they all maintained high aerobic capacity and recovered rapidly from exhaustive exercise, even when tested at 3°C above current maximum temperatures. Below, we discuss the management and conservation implications of these results.

### Coastal cutthroat trout in Oregon differ in thermal tolerance

We found compelling evidence that fish from different watersheds (McKenzie, N. Santiam, Alsea, Siletz) differed in physiological performance and thermal tolerance. The fish from the coastal watersheds had higher thermal tolerance (2–3°C higher CT-max) compared with the two watersheds in the Willamette River basin, but fish clearly differed in physiological performance on a finer scale, even between coastal watersheds. Fish from the Alsea watershed clearly demonstrated a decrease in performance with warming: MMR, AAS, FAS and recovery time (Time[MMR_50_]) all decreased to 60–80% of maximum performance in the climate change scenario compared with current ambient temperature conditions. In contrast, fish in the other coastal watershed, Siletz, displayed an increase in performance with warming: AAS and MMR were maximal at the highest test temperature (i.e. Climate treatment). Notably, Alsea had a 23% higher AAS then Siletz but a 1.5°C lower T_pejus_, which suggests there may have been a trade-off between aerobic scope and thermal tolerance. It is possible that fish in the Alsea require a greater aerobic scope and the cost could be reduced thermal breadth for performance. For the Willamette River basin watersheds, the McKenzie fish performed exceptionally well at the climate change temperature, displaying maximal performance for all traits (MMR, AAS, FAS, recovery time). In contrast, the N. Santiam fish did have high AAS and MMR at the warmest test temperature, but FAS and recovery duration were clearly impaired. These results suggest that there are different metabolic responses to acute thermal challenges for fish across these watersheds.

Strong intraspecific variability is beneficial for species resilience to climate change and provides important contributions for humans as well (Des Roches *et al.*, 2021). Similar to the current study, intraspecific variability in physiological performance and thermal tolerance has been reported in numerous salmonid species including sockeye salmon (Lee *et al.*, 2003; Eliason *et al.*, 2011; Chen *et al.*, 2013; Whitney *et al.*, 2016; Anttila *et al.*, 2019), chum salmon (Abe *et al.*, 2019), Chinook salmon (Zillig *et al.*, 2021) and brook trout (Stitt *et al.*, 2014). Our study presents a unique approach by contrasting fish from different hydrologic regimes and thermal histories. Further, understanding this variability for a species with such varied life history, diverse habitat use and broad spatial extent is novel and helps us understand the persistence of phenotypic variation that contributes to the adaptive potential of a population; high variation may enable populations to adapt and persist under novel, new conditions (Hoffmann *et al.*, 2017). Further, phenotypic diversity enables the portfolio effect, where detrimental changes in environmental conditions are buffered by phenotypic variation across populations such that the aggregated overall population remains stable (Brennan *et al.*, 2019; Schindler *et al.*, 2010). Thus, across systems, intraspecific variation should be evaluated, maintained and restored to support healthy and resilient populations.

### Coastal cutthroat trout in Oregon are at low risk of thermal stress

We found no evidence that coastal cutthroat trout from any of the watersheds suffered from a substantial reduction in performance across the temperature range tested here. The temperatures tested were meant to reflect currently observed diurnal variability, maximum temperature and climate warming. In this way we attempted to summarize and describe not only the physiological demands associated with daily thermal cycling but also how phenotypic traits that characterize metabolism operate under thermal stress (Halsey *et al.*, 2018). However, we found no evidence of a major metabolic constraint as temperatures warm. AAS did not decrease below 70% of maximum levels at any of the test temperatures for any of the watersheds (Fig. S3). While FAS did decrease in the Climate treatment for most of the watersheds, a high FAS (i.e. >3) was still maintained across temperatures, which suggests fish would have had ample energy available to be able to digest a meal, swim, escape predators, etc. (Eliason *et al.*, 2008; Eliason *et al.*, 2022; Farrell, 2016). This finding is supported by the recovery performance. Across all temperatures, fish rapidly recovered from the chase treatment and were back to 50% of their MMR level within 10–25 min. Although recovery duration did significantly increase with the Climate treatment, functionally, this only amounted to an additional ~5 min of recovery time. This suggests the fish would be able to resume normal activities within 10–15 min of an exhaustive exercise event, across any of the temperatures tested here. Similarly, maintenance metabolism did not become a loading factor on aerobic scope as temperature warmed. Specifically, while RMR did increase as temperatures warmed, it did so at a modest rate (i.e. Q10 values were below 3.5). Other studies have similarly found that some salmonids can maintain high physiological performance across the range of encountered temperatures, including Chinook salmon (Poletto *et al.*, 2017) and rainbow trout *Oncorhynchus mykiss* (Verhille *et al.*, 2016).

### TSMs and functional WT

One actionable approach to understanding species thermal needs and risk is to evaluate thermal tolerance in relation to environmental temperatures. We calculated TSM using CT-max values together with stream temperature data to get a depiction of relative tolerances for fish across these four watersheds. TSM values were highest for the Alsea fish when compared with fish in the other watersheds. The fish in the McKenzie watershed, which reside in streams that currently have cool, stable thermal regimes, had the second highest and most variable TSM values, which contrast with their overall metabolic performance. Research by Sandblom *et al.* (2016) may explain this discrepancy. They found that while basal energy requirements and resting cardiorespiratory functions are thermally plastic, maximum capacities and upper critical heat tolerances are much less flexible and will limit the adaptive capacity of fish as the climate warms. Using 2080 projections of stream temperature, all TSMs decreased as predicted, but most still showed a strong buffer before lethality (3–5°C).

Evaluating TSM values can provide an approach for managers to identify and broadly categorize populations that may be at risk (Walters *et al.*, 2018). However, individuals will experience significant thermal stress at temperatures below their thermal maximum, which will inhibit performance and productivity (Blasco *et al.*, 2020). WT values, the difference between T_pejus_ and environmental temperatures, can provide a more actionable metric for managers as it is more ecologically relevant. Here, we propose a new threshold for T_pejus_ for subadult salmonids based on a threshold of FAS (FAS = 3). FAS can indicate when a metabolic constraint is developing for fish, specifically when baseline metabolism costs become prohibitively high (see Material and Methods for thorough description). The Siletz fish had the highest T_pejus_ reflecting the inherently warm conditions they experienced. The Alsea and N. Santiam fish had similar WT (~ 7.5°C) despite different T_pejus_ and thermal histories. As such, these fish in these watersheds do not appear to be at immediate threat from thermal stress. Additionally, these species appear well adapted to their local conditions while also have similar ‘buffers’ to climate change warming. We sampled low elevation, coastal and moderate to higher elevation headwater streams to display the breadth of thermal variability coastal cutthroat trout experience across their range in Oregon. Whether we could apply similar WT values across their full geographical range (i.e. Alaska to Northern California) based on their thermal history is still an open question. Exploring T_pejus_ variability given potentially similar underlying environmental conditions influencing thermal regimes and contrasting T_pejus_ for species across distributions could elucidate how species are able to tolerate and buffer the short-term consequences of temperature increases.

### Data needed to move the field forward and assist managers

The present study found compelling evidence that acute thermal sensitivity varies across watersheds and matches thermal history; however, we cannot wholly conclude that these differences are due to local adaptation. While we took tissue samples from the fish for potential future genetic analysis, the genetic variation within or between watersheds is currently unknown. Furthermore, a common garden approach is necessary to examine adaptive variation, which was not used here. It is possible that the results obtained here could be primarily due to phenotypic plasticity (i.e. field acclimatization processes experienced by the fish in their local environments). However, given the strong evidence of local adaptation among populations of salmonids (Adkison, 1995; Fraser *et al.*, 2011), including cutthroat trout (Drinan *et al.*, 2012), it is also possible that a genetic x environment interaction is an important mechanism underlying our trends. Future interdisciplinary work examining the adaptive capacity of cutthroat trout to climate change is warranted.

More broadly, improvements in physiological thermal sensitivity data across life stages and range of freshwater fish species will provide a strong understanding of their ability to tolerate climatic variation and provide managers clues on how to mitigate climate thermal stress. Many studies have shown that knowledge of life stage and life history traits (e.g. reproductive rates, dispersal abilities, physiological tolerances, etc.) that inform sensitivity can be as useful for understanding of taxonomy and distribution (Dahlke *et al.*, 2020; Pacifici *et al.*, 2015; Willis *et al.*, 2015).

Finally, understanding current thermal exposure for stream-dwelling species is challenging given the lack of continuous stream temperature data that captures and characterized stream habitats at spatial (river basins, streams, coastal to inland, etc.) and temporal scales (daily, seasonal, annual) (Fitz Gerald *et al.*, 2021). Improvements in the consistency, spatial and temporal interval of sampling and access to stream temperature data will improve the accuracy of WTs.

### Advantages and limitations of the approach

Stream-side respirometry provides a direct link between phenotypic trait expression and environmental temperatures. Stressors are reduced (fish handling and transportation are minimized, natal water source is utilized) and fish are returned to the same location in the stream where they were captured. We are able to evaluate metabolic trait expression under naturally fluctuating conditions throughout a diel cycle, which is not typically possible in a laboratory setting. There are, however, trade-offs to field- versus laboratory-based respirometry and some issues to consider when evaluating field-based results. One logistical constraint with the field respirometry method can be reliable and consistent daily access to fish. In general, our fish were similarly sized and most (>80%) were less than 152 mm though, it is possible that some individuals were at different stages of sexual reproduction, which may have contributed to some of the variability in our data. However, we found no differences in any of the metabolic traits evaluated as a result of length or mass. Further, it is also conceivable that some of the fish from the coastal watersheds were migrants (versus residents). This may mean that metabolic costs were partitioned differently and may have contributed to increased variation in MO_2_ in those watersheds.

A second constraint is related to fish capture in a field setting. In our study, we used electrofishing to capture fish. Unfortunately, there is not a huge body of literature on recovery dynamics from electrofishing. We certainly would anticipate that electrofishing would have behavioural and physiological impacts on the fish immediately after capture. For example, Schreer *et al.* (2004) demonstrated that rainbow trout were behavioural impaired for up to 1 h after electroshock and cardiac function took 2–3 h to return to resting levels. A study with cutthroat trout found that plasma lactate and cortisol increased in response to electroshocking but returned to baseline levels by 6 h (Mesa and Schreck, 1989). Given these two studies, we expect the fish were likely recovered by the time the experiments began, but we cannot be certain.

A third consideration in this study is that fish were only exposed to ecologically relevant, brief overnight thermal acclimation periods, so full thermal acclimation processes were likely incomplete; however, fish were already acclimatized to summer temperatures (experiments were conducted in August). Thus, caution must be used when comparing these results with other laboratory-based studies where fish were laboratory acclimated for many weeks to a thermal regime. Fish may not have been entirely postprandial when the experiment began (18 h after capture) if they had consumed a meal immediately before capture and this could have influenced the results with slightly higher metabolic costs for fish still digesting.

Despite the constraints highlighted above, these data have wide applicability from helping to define parameters in bioenergetics models (e.g. optimal temperatures at which aerobic scope is maximized), to providing an understanding of movement and survival strategies among individuals from fragmented habitats (Armstrong *et al.*, 2021; Hahlbeck *et al.*, 2021). Utilizing field data describing both stream temperatures and species physiological tolerances improves the accessibility of these data for managers who want to make use of the best available science but need translatable measures on which to base decisions and actions.

### Summary and conclusions

This study provides a promising and hopeful outlook for Coastal cutthroat trout in terms of their vulnerability to climate changes that will likely alter thermal regimes. Coastal cutthroat trout displayed considerable intraspecific variability in physiological performance and thermal tolerance across the four watersheds we examined. Thermal tolerance matched the historical experience: the coastal watersheds experiencing warmer ambient temperatures had higher critical thermal tolerance compared with the interior, cooler Willamette watersheds. Physiological performance varied across all four watersheds. There was evidence of a trade-off between high aerobic performance and broad thermal tolerance. The fish with the highest aerobic scope (Alsea) displayed a decline in aerobic scope with warming and lower T_pejus_, while fish from a neighbouring watershed (Siletz) maintained a consistent, albeit lower aerobic scope across the full range of test temperatures and higher T_pejus_. This high intraspecific variability is anticipated to confer strong adaptive capacity for the species. Notably, all these Coastal cutthroat trout populations appear to be at low risk of thermal stress. Ambient water temperature could warm a further 5–7°C before functional performance is expected to become impaired. Finally, this study provides a practical framework for future studies on intraspecific variability in freshwater fishes using a novel stream-side respirometry system.

## Data Availability Statement

Data are not yet provided but will be available upon acceptance of this manuscript. R code to analyse metabolic rate performance metrics will be open source on Github (all links to source code and complementary documentation will be provided after revisions). No other novel code was used. Data will be permanently archived here: https://github.com/kjadunn/Coastal-Cutthroat-Trout-Respo/.

## Supplementary Material

Web_Material_coac029
