# Supplementary material for: Intraspecific variability in thermal tolerance: a case study with coastal cutthroat trout
Source: Conserv Physiol. 2022 May 12;10(1):coac029. doi: 10.1093/conphys/coac029 (PMC9178963; doi:10.1093/conphys/coac029)
Supplement: Web_Material_coac029 [file web_material_coac029.zip › Supplemental figures and tables ver9.docx]

**Supplementary Tables and Figures**


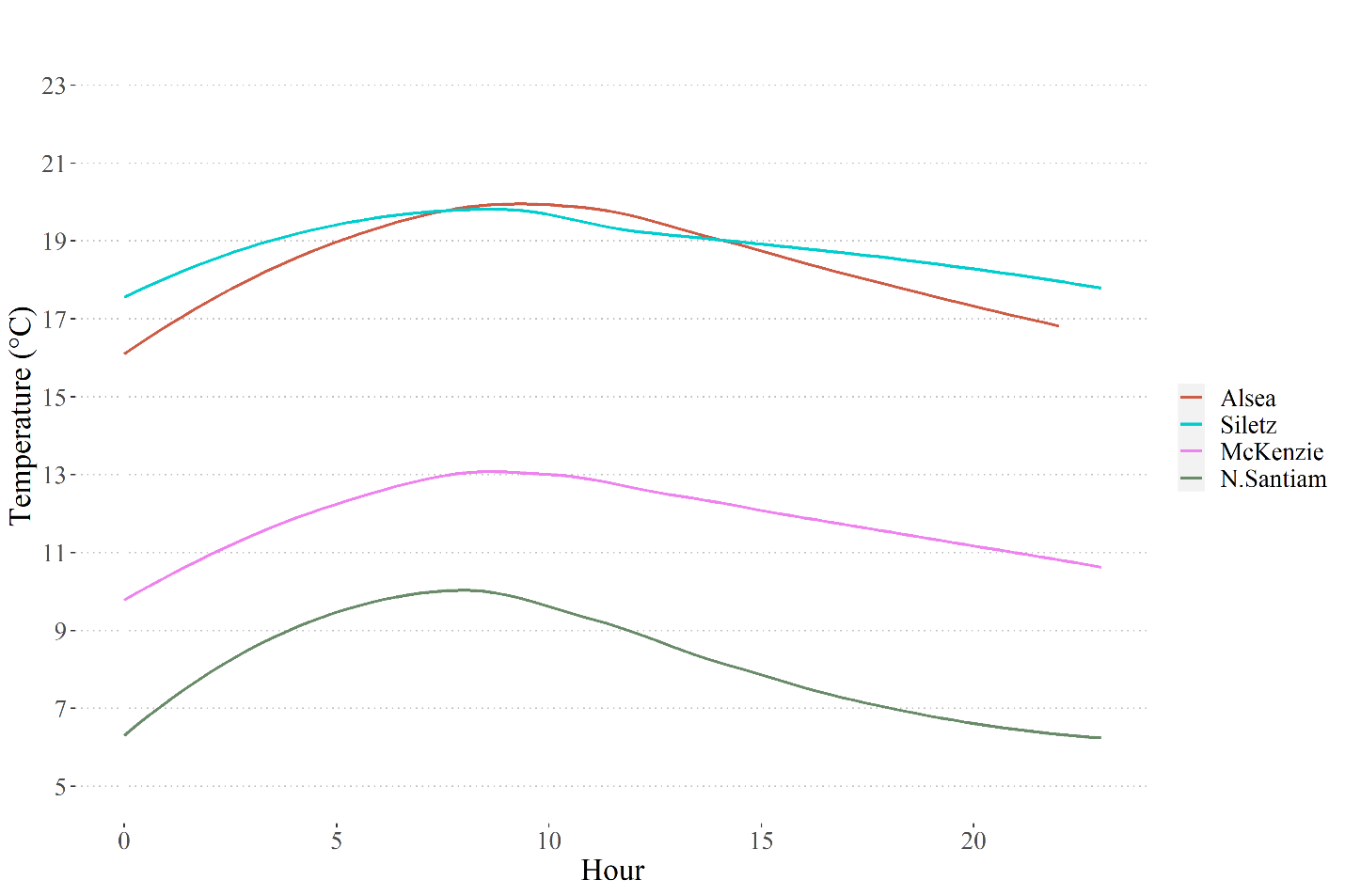


Figure S1. Temperature profiles from ambient temperature trials in each watershed. Color corresponds to watershed.


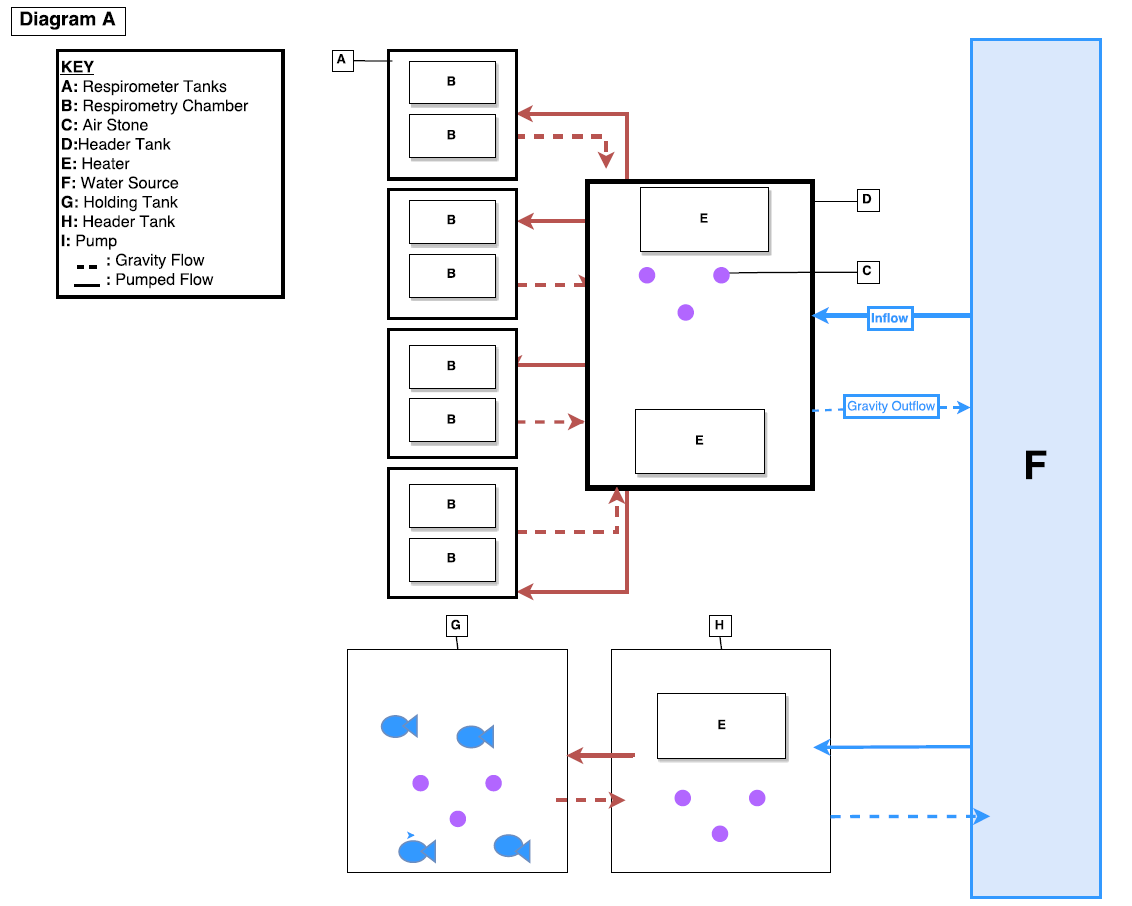


Figure S2. Design and set-up of respirometry system.


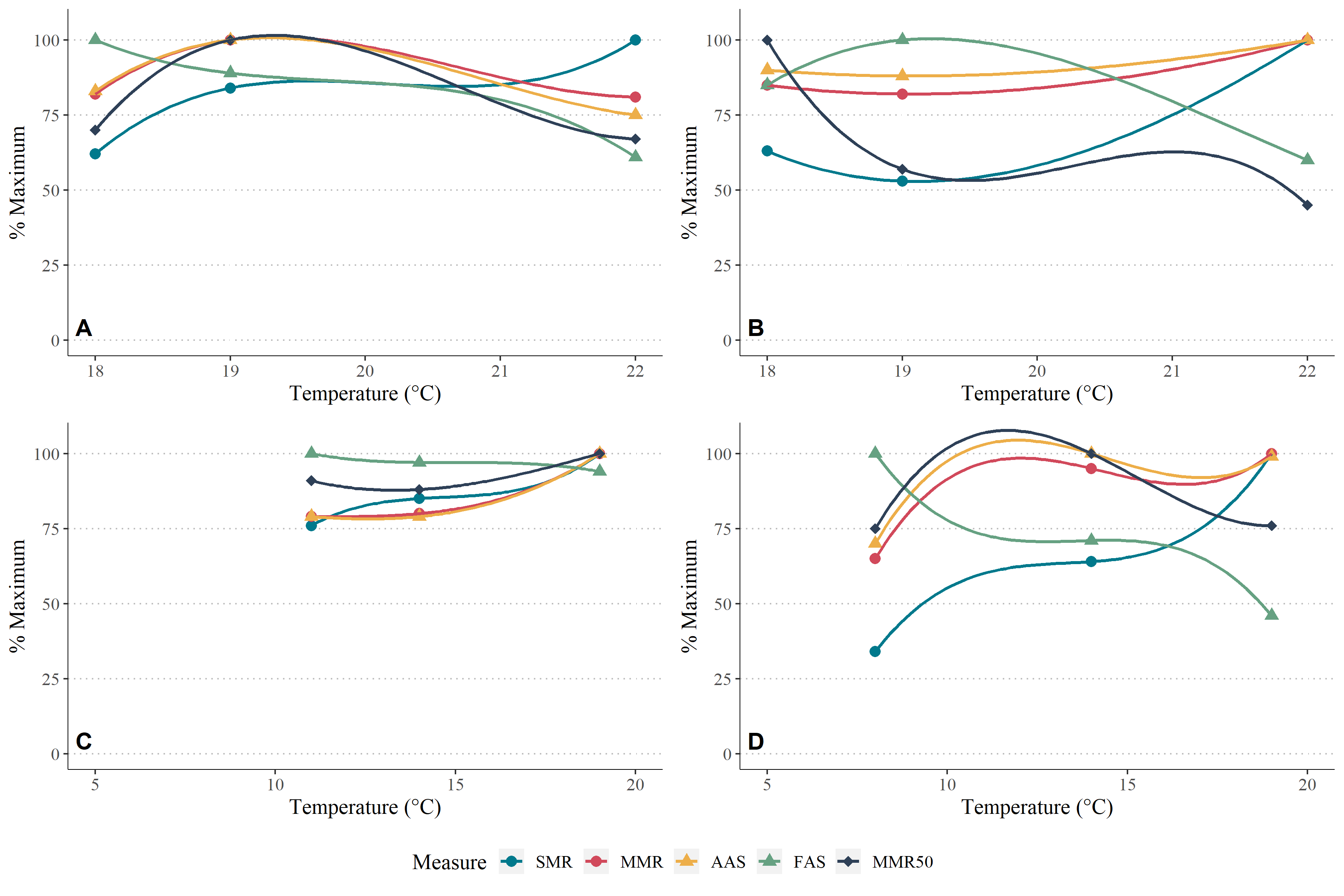


Figure S3. Percent of maximum value for all metabolic measures for the (A) Alsea, (B) Siletz, (C) McKenzie, and (D) North Santiam. SMR = standard metabolic rate, MMR = maximum metabolic rate, AAS = absolute aerobic scope, FAS = factorial aerobic scope, and MMR50 = the time (min) it took for the fish to recover to 50 % of their MMR after exhaustive exercise.


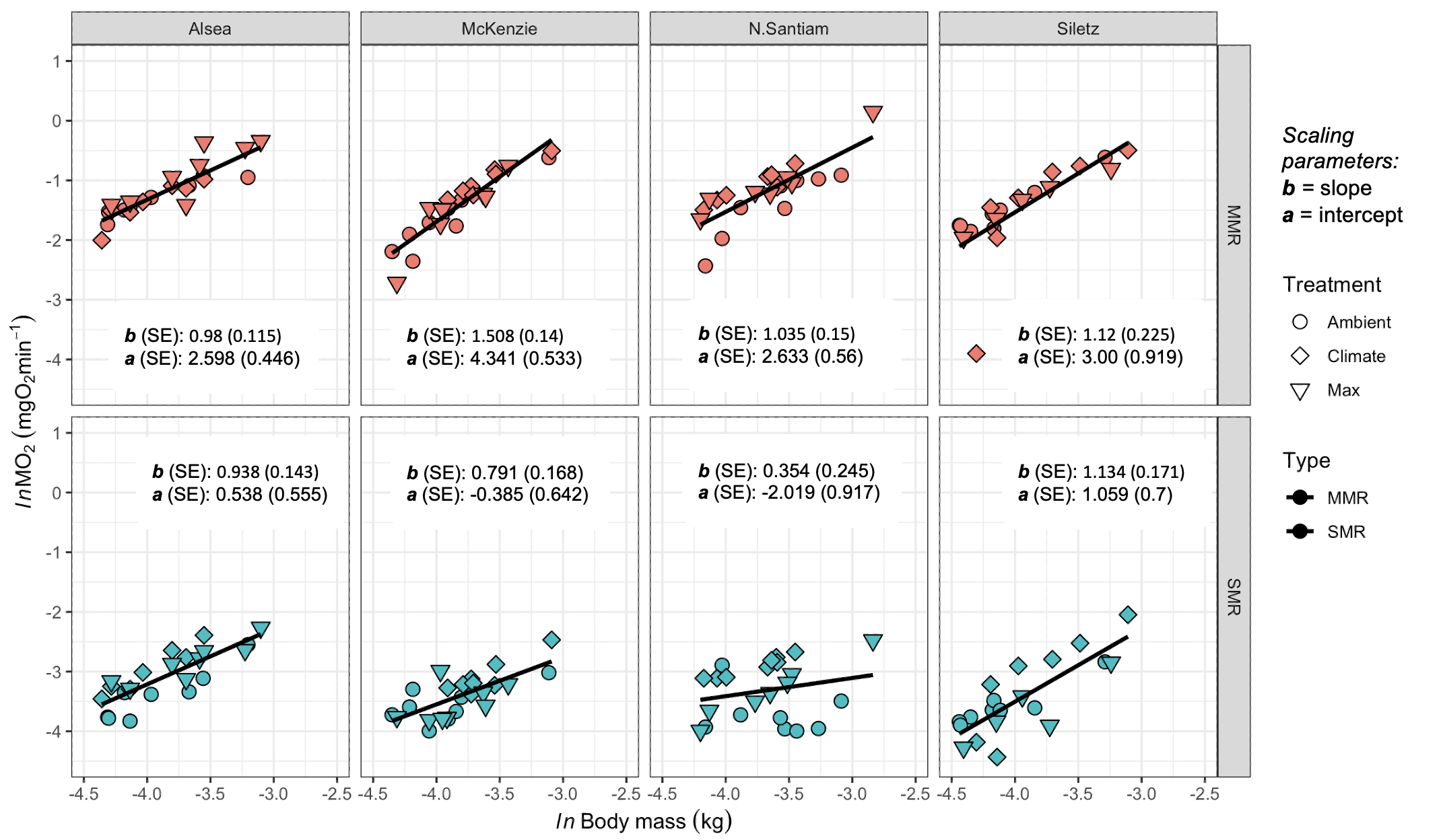


Figure S4. Body mass scaling relationships of maximum metabolic rates (MMR) and standard metabolic rates (SMR). Plotted are simple linear regressions of plotted data. Each dot is metabolic rate (MMR = red and SMR = blue) measurement of individual fish. SE = standard error. The absolute size range of all fish together was 8.9 - 58.6 grams.


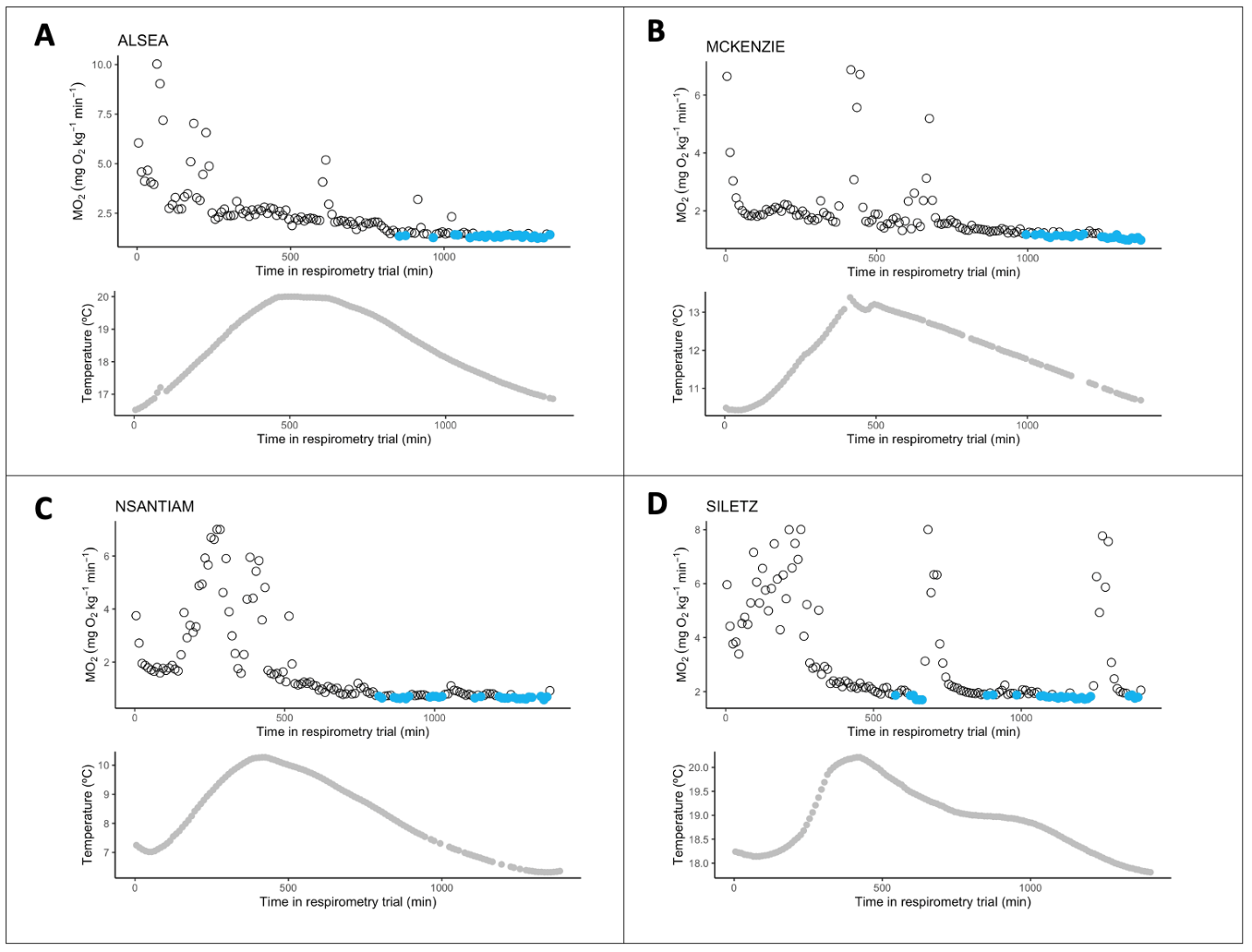


Figure S5. Four representative traces of fish metabolic rates overnight; one for each Basin, (A) Alsea, (B) McKenzie, (C) N. Santiam, and (D) Siletz Basin). In each panel the top plot shows metabolic rate measurements overnight, the bottom plot with grey traces show temperature (º C) over the same time span.

Table S1. Mean and standard error (SE) of body mass, length, and condition factor for all coastal cutthroat trout individuals.

Basin Temperature Stream Body weight SE Fork Length SE Condition SE Trial (g) (mm) Factor*

Alsea Ambient Cove Cr 21.46 3.37 126.1 6.8 1.02 0.04
 Max Unnamed Trib 27.25 3.79 138.8 6.0 1.00 0.10
 Climate Carns Canyon Cr 19.46 2.11 121.8 5.0 1.05 0.02

Siletz Ambient Little Rock Cr 17.80 2.99 121.8 6.5 1.13 0.25
 Max Little Rock Cr 17.50 3.60 120.6 8.7 0.93 0.03
 Climate Little Rock Cr 21.75 3.98 128.3 7.2 0.96 0.02

McKenzie Ambient White Branch Cr 21.06 3.55 126.0 5.9 1.00 0.02
 Max White Branch Cr 21.81 2.19 127.5 4.7 1.03 0.03
 Climate White Branch Cr 27.39 2.84 140.5 4.8 0.97 0.03

N. Santiam Ambient N. Santiam R 28.40 3.64 142.5 6.9 0.95 0.03
 Max N. Santiam R 26.14 5.34 134.5 8.9 0.98 0.03
 Climate N. Santiam R 23.65 2.08 133.6 4.5 0.97 0.02

*Fulton Condition Factor: *K* = 100,000*W*/*L*^3^

*where:* W= the weight of the fish in grams;
 L = the fork length of fish in millimeters.

Table S2. Critical thermal maximum (CT max), thermal safety margin (TSM), temperature at which FAS ~ 3 (T_pejus_) and functional warming tolerance (WT) values. NorWeST historical (2002-2011) Maximum Weekly Maximum Temperature (MWMT) and the 2080 A1B projections of MWMT (from the streams where fish were collected) were used to calculate TSM and WT. Mean and standard error displayed. Results of the Dunn’s Multiple Comparison Test provided by grp. Different letters indicate significant pairwise differences. We did not calculate T_pejus_ for the McKenzie fish; therefore WT values were not calculated for that location.

Location CT max grp n Thermal Safety Margin T_pejus_ Functional Warming Tolerance

Current grp 2080 Current 2080

Alsea 29.7± 0.1 a 8 12.8 ± 0.1 a 11.0 ± 0.1 24.5 7.5 5.7

Siletz 30.3 ± 0.1 a 8 11.5 ± 0.1 ab 9.6 ± 0.1 27.0 8.1 6.2

McKenzie 27.2 ± 0.9 b 8 11.6 ± 0.9 ab 9.9 ± 0.9 N/A N/A N/A

N. Santiam 27.2 ± 0.2 b 8 11.5 ± 0.3 b 9.8 ± 0.3 23.5 7.7 5.9

Table S3. Modelled relationships between factorial aerobic scope (FAS), absolute aerobic scope (AAS), and average study temperatures for each watershed. Linear regression models where aerobic scope (AAS or FAS) = intercept + slope + error.

| Basin | N | FAS model parameters | | AAS model parameters | | **Predicted T_pejus_ (ºC), where FAS = 3** |
| --- | --- | --- | --- | --- | --- | --- |
|  |  | *Slope (SE)* | *Interc. (SE)* | *Slope (SE)* | *Interc. (SE)* |  |
| Alsea | 23 | -0.828 (0.236) * | 23.564 (4.745) | -0.701 (0.474) | 26.327 (9.354) | 24.5 |
|  |  |  |  |  |  |  |
| Siletz | 22 | -0.704 (0.316)* | 21.945 (6.241) | 0.37 (0.204) | 4.766 (4.038) | 27.0 |
|  |  |  |  |  |  |  |
| N. Santiam | 23 | -0.683 (0.151)* | 19.201 (2.238) | 0.304 (0.117) | 6.952 (1.74)* | 23.5 |
|  |  |  |  |  |  |  |

* Significant relationships p < 0.05

Table S4. Model summary output describing RMR changes across temperatures in coastal cutthroat trout. Temperature ranged from 6 to 22 ºC across all fish combined. Each individual fish had RMR at minimum of 3 temperatures and maximum of 8. Estimates below are maximum likelihood estimates using mixed effect model. Model Formula: *ln*(MO_2_) ~ Temperature + watershed - 1 + (1 | FishID).

| **Main Effects:** | | |  | |  |  |
| --- | --- | --- | --- | --- | --- | --- |
|  | *Coefficient (β)* | *SE* | | | *Confidence Intervals* | *Exponential form:* |
| **Temperature** | 0.073 | 0.007 | | | 0.059 – 0.088 |  |
| **Alsea** | -0.574 | 0.160 | | | -0.891 – -0.257 | RMR ~ 0.5628 + 1.0765^Temperature^ + ε |
| **McKenzie** | -0.478 | 0.130 | | | -0.736 – -0.219 | RMR ~ 0.6198 + 1.0765^Temperature^ + ε |
| **N. Santiam** | -0.404 | 0.108 | | | -0.618 – -0.186 | RMR ~ 0.6675 + 1.0765^Temperature^ + ε |
| **Siletz** | -0.592 | 0.163 | | | -0.915 – -0.269 | RMR ~ 0.5529 + 1.0765^Temperature^ + ε |
| **Random (intercept) effects:** | | | | |  |  |
|  | *Variance* | *SD* | | |  |  |
| **FishID** | 0.029 | 0.170 | | |  |  |
| ***Residual*** | 0.079 | 0.282 | | |  |  |
| N = 157, Random effects: FishID (N=32); ε = error | | | |  | | |

Table S5. Mean and SEM for standard metabolic rate (SMR) and maximum metabolic rate (MMR) for coastal cutthroat by location and treatment. The mean temperature represents the average temperature during which the lowest 20% of MO2 values were measured (i.e. when SMR was measured). The temperature at the time of the chase is also displayed and corresponds to the MMR values. For all location and temperature treatment, N = 8 individuals. Kruskal-Wallis chi-squared test (K-W) results with post-hoc Dunn’s multiple comparison test denoted by letters corresponding to treatment (grp).

Location Treatment Temperature (°C) SMR MMR

Mean Chase MO_2_ SEM Grp K-W P value MO_2_ SEM Grp K-W P value

Alsea 14.591 0.000 7.417 0.024

Ambient 18.6 16.5 1.718 0.107 a 13.755 0.695 x

Max 19 19.3 2.314 0.147 b 16.798 1.391 y

Climate 22 22.1 2.755 0.121 b 13.659 0.626 x

Siletz 14.04 0.000 9.690 0.007

Ambient 18.8 18.1 1.683 0.075 a 13.493 0.499 x

Max 19 19.9 1.425 0.129 a 12.936 0.400 y

Climate 22 22.3 2.232 0.291 b 13.148 1.949 y

McKenzie 6.952 0.030 8.757 0.012

Ambient 11.8 10.5 1.512 0.176 a 9.865 0.732 x

Max 14 14.3 1.452 0.188 a 10.867 1.025 x

Climate 19 19.5 1.716 0.076 a 13.568 0.335 y

N. Santiam 17.615 0.000 12.99 0.001

Ambient 8.6 7.4 1.094 0.304 a 9.336 0.776 x

Max 14 14.1 1.521 0.122 b 13.955 1.025 y

Climate 19 19.2 2.371 0.096 c 14.631 0.471 y

* MO_2_ (mg O_2_ kg^-1^ min^-1^) = Mass-specific oxygen consumption rate.

Table S6. Mean and SEM for absolute aerobic scope (AAS) and factorial aerobic scope (FAS) for coastal cutthroat by watershed and temperature trial. The mean temperature represents the average temperature during which the lowest 20% of MO2 values were measured (i.e. when SMR was measured). For all location and temperature treatment, N = 8 individuals. Kruskal-Wallis chi-squared test (K-W) results with post-hoc Dunn’s multiple comparison test denoted by letters corresponding to treatment (grp).

Location Treatment Temperature (°C) AAS FAS

Mean MO_2_ SEM Grp K-W P value MO_2_ SEM Grp K-W P value

Alsea 8.834 0.012 11.209 0.003

Ambient 18.6 12.037 0.719 a 8.246 0.678 a

Max 19 14.484 1.320 ab 7.302 0.526 a

Climate 22 10.904 0.580 a 4.990 0.231 b

Siletz 2.888 0.235 9.966 0.006

Ambient 18.8 11.810 0.545 a 8.197 0.597 a

Max 19 11.511 0.377 a 9.661 1.059 a

Climate 22 10.915 1.735 a 7.533 1.177 b

McKenzie 6.952 0.031 0.931 0.627

Ambient 11.8 8.353 0.865 a 7.326 1.062 a

Max 14 9.415 1.123 a 8.347 1.172 a

Climate 19 11.851 0.368 b 8.055 0.531 a

N. Santiam 9.642 0.008 10.052 0.006

Ambient 8.6 8.242 0.931 a 12.025 2.250 a

Max 14 12.434 1.022 b 9.462 0.841 a

Climate 19 12.260 0.457 b 6.224 0.274 b

* MO_2_ (mg O_2_ kg^-1^ min^-1^) = Oxygen consumption rate.

Table S7. Mean and SE MO_2_ values at shared 19⁰ C temperature for standard metabolic rate (SMR), maximum metabolic rate (MMR), absolute aerobic scope (AAS), and factorial aerobic scope (FAS). Kruskal-Wallis chi-squared test (K-W) results with post-hoc Dunn’s multiple comparison test denoted by letters corresponding to treatment (grp).

Measure Location Mean SE grp K-W P value
 Chi-squared

SMR 20.645 0.000

Alsea 2.314 0.147 a

 Siletz 1.425 0.129 b

 McKenzie 1.716 0.076 b

 N. Santiam 2.371 0.096 a

MMR 13.636 0.003

Alsea 16.798 1.391 a

 Siletz 12.936 0.400 b

 McKenzie 13.568 0.335 b

 N. Santiam 14.631 0.471 b
AAS 10.432 0.015

Alsea 14.484 1.320 a

 Siletz 11.511 0.377 b

 McKenzie 11.851 0.368 b

 N. Santiam 12.260 0.457 b

FAS 16.102 0.001

Alsea 7.302 0.526 a

 Siletz 9.661 1.059 b

 McKenzie 8.055 0.531 b

 N. Santiam 6.224 0.274 a

MMR50 (minutes) 6.415 0.093

Alsea 13.250 1.346 a

 Siletz 11.0 0.681 a

 McKenzie 11.875 0.743 a

 N. Santiam 13.0 0.378 a

Table S8. For each treatment, these data represent the time (Minutes) it takes to get to 50% of an individual fish’ maximum metabolic rate (MO_2_) [MMR50]. Kruskal-Wallis chi-squared test (K-W) results with post-hoc Dunn’s multiple comparison test denoted by letters corresponding to treatment (grp).

Location Treatment n MO_2_ SE Minutes SE grp K-W P value
 chi-squared

Alsea 8.135 0.017

Ambient 8 36.242 4.985 13.875 2.949 a

Max 8 41.421 4.096 13.250 1.346 a

Climate 7 47.635 4.151 19.714 2.542 b

Siletz 4.914 0.085

Ambient 8 33.193 5.164 24.375 13.104 a

Max 8 28.073 1.476 11.000 0.681 a

Climate 6 41.921 1.440 14.000 1.095 a

McKenzie 0.047 0.976

Ambient 6 28.348 3.816 12.333 1.256 a

Max 8 26.729 1.909 13.500 2.428 a

Climate 8 31.570 2.217 11.875 0.743 a

N. Santiam 7.961 0.018

Ambient 7 18.332 1.400 9.714 0.644 a

Max 8 25.483 4.897 9.875 1.315 a

Climate 8 39.906 1.411 13.000 0.378 b

Table S9. Metabolic scaling relationships and range of body mass (kg) in each Treatment. The metabolic scaling coefficients were generated from linear regression between *ln*MO2 (dependent variable) and *ln*Body mass (independent variable). Standard error (SE) is provided for each estimate.

| Basin | Metabolic rate measurement | Metabolic scaling slope (SE) | Metabolic scaling intercept (SE) | Sample size (n) | Min body mass (kg) | Max body mass (kg) |
| --- | --- | --- | --- | --- | --- | --- |
| **Alsea** | **MMR** | **0.98 (0.115)** | **2.598 (0.446)** | **24** | 0.0128 | 0.0449 |
|  | *Ambient* | 0.693 (0.103) | 1.408 (0.403) | 8 |  |  |
|  | *Climate* | 1.058 (0.197) | 2.836 (0.784) |  |  |  |
|  | *Max* | 0.985 (0.251) | 2.74 (0.927) |  |  |  |
|  | **SMR** | **0.938 (0.143)** | **0.538 (0.555)** | **24** |  |  |
|  | *Ambient* | 0.976 (0.174) | 0.432 (0.686) | 8 |  |  |
|  | *Climate* | 1.227 (0.15) | 1.909 (0.597) |  |  |  |
|  | *Max* | 0.746 (0.15) | -0.107 (0.552) |  |  |  |
| **McKenzie** | **MMR** | **1.508 (0.14)** | **4.341 (0.533)** | **24** | 0.0129 | 0.0455 |
|  | *Ambient* | 1.324 (0.205) | 3.541 (0.811) | 8 |  |  |
|  | *Climate* | 1.035 (0.115) | 2.732 (0.418) |  |  |  |
|  | *Max* | 1.779 (0.356) | 5.351 (1.379) |  |  |  |
|  | **SMR** | **0.791 (0.168)** | **-0.385 (0.642)** | **24** |  |  |
|  | *Ambient* | 0.495 (0.257) | -1.616 (1.017) | 8 |  |  |
|  | *Climate* | 1.035 (0.217) | 0.661 (0.787) |  |  |  |
|  | *Max* | 0.543 (0.393) | -1.436 (1.519) |  |  |  |
| **N.Santiam** | **MMR** | **1.035 (0.15)** | **2.633 (0.56)** | **24** | 0.0096 | 0.0586 |
|  | *Ambient* | 1.327 (0.244) | 3.392 (0.889) | 8 |  |  |
|  | *Climate* | 0.891 (0.142) | 2.266 (0.537) |  |  |  |
|  | *Max* | 1.108 (0.135) | 3.026 (0.514) |  |  |  |
|  | **SMR** | **0.354 (0.245)** | **-2.019 (0.917)** | **24** |  |  |
|  | *Ambient* | -0.265 (0.392) | -4.674 (1.426) | 8 |  |  |
|  | *Climate* | 0.625 (0.075) | -0.558 (0.282) |  |  |  |
|  | *Max* | 0.817 (0.126) | -0.293 (0.482) |  |  |  |
| **Siletz** | **MMR** | **1.12 (0.225)** | **3 (0.919)** | **24** | 0.0089 | 0.0447 |
|  | *Ambient* | 1.057 (0.117) | 2.832 (0.484) | 8 |  |  |
|  | *Climate* | 1.522 (0.693) | 4.442 (2.741) |  |  |  |
|  | *Max* | 0.936 (0.066) | 2.291 (0.276) |  |  |  |
|  | **SMR** | **1.134 (0.171)** | **1.059 (0.7)** | **24** |  |  |
|  | *Ambient* | 0.816 (0.122) | -0.243 (0.502) | 8 |  |  |
|  | *Climate* | 1.448 (0.403) | 2.47 (1.594) |  |  |  |
|  | *Max* | 0.859 (0.211) | -0.263 (0.885) |  |  |  |
